# Strategies of German Bundesliga and English Premier League clubs for the COVID-19 crisis: the case of international broadcasting fans

**DOI:** 10.1007/s11846-021-00515-3

**Published:** 2022-01-13

**Authors:** José F. Navarro-Picado, Eduardo Torres-Moraga, Manuel Alonso Dos Santos, Brandon Mastromartino, James J. Zhang

**Affiliations:** 1 School of Business Administration, Universidad de Costa Rica, San José, Costa Rica; 2 School of Economic and Business, Universidad de Chile, Santiago, Chile; 3 Marketing and Market Research Department, Universidad de Granada, Granada, Spain; 4 Department of Management, Faculty of Economics and Business Administration, Universidad Católica de la Santísima Concepción, Concepción, Chile; 5 Department of Applied Physiology & Sport Management, Simmons School of Education & Human Development, Southern Methodist University, Dallas, Texas USA; 6 Department of Kinesiology, The University of Georgia, Athens, GA USA

**Keywords:** Legitimacy perception, Decision justifiability, Organizational trustworthiness, Multimedia consumption, M39 marketing and advertising—Other, M59 personnel economics—other

## Abstract

During the COVID-19 pandemic that paused sports worldwide, the German Bundesliga League (GBL) and English Premier League (EPL) took two different strategic approaches to agree with their players on returning to play. To become better informed and prepared for future crisis management, this study examines consumer responses to these opposing strategies. We also identify how perceived organizational legitimacy, trustworthiness, reliance, and justifiability have an impact on consumer multimedia consumption of the games. A sample of 503 participants responded to an online questionnaire regarding the contrasting decisions taken by the GBL and the EPL during the global health crisis. SEM with multi-group analysis was conducted to test the research hypotheses. When comparing the two selected sport leagues, the league that reached an agreement with their players experienced higher levels of perceived legitimacy while needing fewer perceptions of trustworthiness, reliance, and justifiability to obtain higher multimedia consumption intention from consumers.

## Introduction

March 2020 will always be remembered as the month when sporting events, along with many other organized activities and public gatherings, were restricted or canceled worldwide to counteract the spread of COVID-19 (Majumdar and Naha 2020). Although health measures such as the shutdown of stadiums or postponing of games were to protect fans and athletes from contracting the virus (Corsini et al. 2020) and related risks (Mann et al. 2020), the sport industry has faced immense economic pressure to return to normal operations as soon as possible (Mastromartino et al. 2020; Drewes et al. 2021). During crisis periods, such as the COVID-19 era, judging what is normal and what is not is a challenge for society in general (Clark et al. 2020), especially in sport organizations (Hammerschmidt et al. 2021a, b). Among these uncertainties, reliance is an important tool for consumers to maintain an interest in institutions (Doney and Cannon 1997).

Individuals tend to be tolerant of certain changes but simultaneously expect to see an alignment of the new happenings to their relevant institutions (Ödlund 2010). For example, during the COVID-19 crisis, fans expected that sport leagues could reach salary agreements with their players in a way that to allow the team to adjust to the financial issues of the clubs while keeping the players and staff employed (Sheptak and Menaker 2020). In this context, consumers’ evaluations of the legitimacy, justifiability, and trustworthiness of a league’s decisions come into play. Although individual judgments on what is legitimate, justifiable, and dependable have been overlooked in the past, little is known about how these judgments adapt during crisis periods and how they interact with consumption behaviors. As these variables can potentially positively impact consumer evaluation in different contexts, they are deemed relevant for consumption intention behavior (e.g. Sen and Morwitz 1996; Niemi and Kantola 2018; Dabbous and Tarhini 2019; Ismagilova et al. 2020).

It is highly possible that during periods of crisis, perceived organizational legitimacy, trustworthiness, reliance, and the justifiability of the decisions taken by the relevant institutions would show different intensities in individuals’ intentions to consume multimedia. Specifically, this study was based on the social judgment theory (SJT) (Bitektine 2011) and norm theory (NT) (Feldman et al. 2020) and in the context of the COVID-19 health crisis. It was designed to investigate whether institutions that took certain consumer-expected concrete actions would experience higher levels of organizational legitimacy while relying less on the trustworthiness, reliance, and justifiability of a decision to adopt their pathway toward multimedia consumption intention (MMCI). MMCI is deemed to be a relevant variable for professional sport organizations, especially during the current sanitary crisis. Owing to the impossibility of teams and leagues to receive fans in their properties, it is the major and, momentarily, the only exposure channel on matchdays.

Sport broadcasts accrue the largest audience of all television shows (Knobloch-Westerwick et al. 2020). With recent innovations in communications and technology (Haynes 2021), sport fans worldwide can experience live events through different paid platforms (Cobbs and Hilton 2012). This has led top-tier sport leagues to receive increased revenue through broadcasting rights (Tamir 2019). Tamir (2019) further suggests that through a personalized experience of simultaneous game-related content, watching games in the arena has become indispensable for fans to a lesser extent. Domestic broadcasting rights are experiencing a slight drop, while international telecasting deals continue to grow (Geey and Harvey 2019).

Football is the most popular sport globally, accounting for over 3.5 billion fans worldwide, while the next sport (cricket) drops to 2.5 billion fans (Das 2021). Specifically, in Latin America, football is unquestionable as the most popular sport (Sotomayor 2020), suggesting that fans in the region are considerably knowledgeable about top-tier leagues and related topics (Ridge 2017). As one of the most-watched leagues worldwide, EPL games are broadcast in every country in Latin America except Cuba (Premier League 2019). The GBL also has full coverage through free-to-air (FC Bayern 2020) or paid platforms (Bertran 2020).

Previous studies have seldom considered these variables when analyzing how the decisions made by sport leagues in a crisis can impact multimedia consumption, and no fans could access the stadiums or arenas at the time of the study (Corsini et al. 2020; Majumdar and Naha 2020). Therefore, this study focused on identifying the differential roles that perceived organizational legitimacy, trustworthiness, reliance, and the justifiability of decisions by sport leagues play to reach an agreement with players and lead to the MMCI of those leagues. Noticeably, this study considered the situational differences between the GBL and the EPL, in which the GBL could reach a salary agreement between the league and players’ union, while the EPL could not.

Regarding the EPL’s salary agreement, it was reported that there was a lack of interest in making any change (Roan 2020). Simultaneously, it has been argued that although the EPL did not reach an agreement, many other industries did not reach labor agreements. However, unlike other industries (Hammerschmidt et al. 2021a, b), due to media exposure and well-known wealth, the EPL received extensive media attention during this period (Kennedy and Kennedy 2021).

The only information that fans had readily available was through the media, especially for fans outside the UK, such as those in Latin America. Therefore, to the fan’s best knowledge, the actual difference between what was done by the EPL and the GBL was that the first one did not reach a salary agreement while the second one did. Specifically, and according to the news, the EPL showed no further interest in reaching an agreement, which was indicated in multiple media sources such as in the following excerpt:

“In a joint statement, the Premier League, EFL, PFA, and League Managers’ Association said they had a ‘constructive meeting’ on Wednesday regarding the challenges facing the game…No decisions were taken with discussions set to continue in the next 48 hours with a focus on several high-profile matters, including player wages and the resumption of the 2019-20 season.” **BBC Sports, April 2020**

“…footballers and clubs are on the receiving end of flak for the delays when it comes to voluntary salary reductions to help their fellow non-playing colleagues…” **Goal, April 2020**

“The British Health Secretary, Matt Hancock, singled out Premier League footballers in a press conference on Thursday, calling on them to take pay cuts to help the economy during the coronavirus crisis…However, there are clubs like Tottenham Hotspur, who have put non-playing staff on furlough without reducing the wages of their players.” **Marca, April 2020**

## Theoretical background

SJT suggests that individuals use a weighting process when evaluating an object (Hoffmann et al. 2019), employing an attitudinal anchor to guide their categorization (Kyle et al. 2004), and allocating the object toward a latitude of acceptance or rejection (Rhine and Severance 1970). Accordingly, individuals use legitimacy as a relevant classification variable when judging an object (Bitektine 2011). Thus, the credibility of the source (Rhine and Severance 1970), individuals’ experience (Brehmer and Brehmer 1988), and social standards (Kyle et al. 2004) play a key role in the assessment made. Within this framework, an assumption made is that individuals have a lack of access to “real” information (Zacharakis and Meyer 1998), are influenced by “majority group” opinions (Van Swol et al. 2018) and have more inflexible positions depending on their ego involvement toward the focal object (Kyle et al. 2004).

Although the evaluation is performed through the SJT lens every time the object arises, the evaluation process is lasting and stable over time (Mao et al. 2018). Regardless of the circumstances, individuals expect established organizations to behave according to their legitimacy as a base element for any decisions. This is especially the case under difficult circumstances, such as the COVID-19 pandemic (Kraus et al. 2020). However, NT is a stimulus-centered judgment, where an individual faces a situation; regardless of past events, they need to allocate the object into a normality categorization (Kahneman and Miller 1986). When evaluating what is normal and what is not, individuals are more affected by abnormal decisions, arising sentiments of regret (Feldman et al. 2020), and contextual situations (Emami et al. 2021) have a relevant impact on the evaluation of whether action or inaction should be considered a more normal decision (Temerak and El-Manstrly 2019). According to Kahneman and Miller (1986), exceptional or out-of-the-routine events are those that seem to have highly available alternatives. Conversely, regular or routine events are sometimes easy representations that evoke little surprise in the judge’s mind. It has been suggested that these situational cues produce common social expectations (Blay et al. 2018), thus guiding public opinion. Blay et al. (2018) argue that individuals cannot separate themselves from their social contexts, creating pressure toward accepting certain social norms and guiding individuals to justify any decision taken to align with these circumstances.

Football fandom, local leagues and clubs, and top-tier football leagues are part of Latin American culture (Sotomayor 2020). People across the continent have access to live or delayed broadcasting of the entire GBL and EPL season games among other premium football leagues (Premier League 2019; Bertran 2020; FC Bayern 2020), fostering a massive, knowledgeable fan base who regularly gather with friends and family to watch games (Ridge 2017).

Noticeably, top-tier professional football leagues, including GBL and EPL, enjoy a loyal base globally and specifically in the Latin American region (Das 2021). Consumers constantly place football media content on top of their broadcasting program`s choice list (Knobloch-Westerwick et al. 2020). Certainly, the high media attention can make the top leagues vulnerable to positive, negative, or unwanted media exposures (Kennedy and Kennedy 2021).

During crisis conditions, organizations regularly show poor efficiency, cooperation (Ödlund, 2010), and coopetition behaviors (Hammerschmidt et al. 2020) while experiencing difficulties in their communication processes (Palttala and Vos 2011). This is why individuals tend to better evaluate institutions that change their normality and proceed with action toward adaptation (Feldman 2020). For example, during the health crises caused by COVID-19, individuals expected organizations to behave according to what was needed in that specific environment and time, such as financial agreements to preserve jobs and protect employees. Within the specific sports context in which we conducted the study, GBL clarified that their top priority was job retention and employee protection (Drewes et al. 2021), indirectly avoiding clubs from entering into higher financial risks (Horky 2021). Simultaneously, EPL was unclear on any action or intention at the moment (Kennedy and Kennedy 2021). The SJT and NT theories help us understand the evaluations made by individuals toward the decisions taken by organizations.

### Perceived organizational legitimacy

Drawing on SJT, legitimacy perception is considered as a baseline foundation that would affect any other upgraded categorization (Bitektine 2011) and is important for institutional survival and eventual thriving (Hutchins et al. 2019). Regarding NT, theorists suggest that moral virtues such as benevolence (trustworthiness) and reliance help facilitate the creation and development of social norms (Blay et al. 2018). As few studies have specifically examined this topic area, little is known about how perceived organizational legitimacy can help or benefit an organization’s subsequent trustworthiness and reliance. Individuals tend to evade regret by adopting more justifiable options (Inman and Zeelenberg 2002), thereby avoiding dubious decisions (van de Calseyde et al. 2018). Simultaneously, people feel a higher need for stability (i.e., sticking to what they have) when the decision-maker is themselves. However, people tend to recommend switching behavior when the object of analysis is someone else (Saine et al. 2018).

When evaluating an institution, the higher the perceived organizational legitimacy of the organization, the higher its trustworthiness, and the easier it is to justify any decision taken by the same organization (Fan and Wu 2019). Within sports, perceived organizational legitimacy can be expected to impact both sponsorship garnering (Navarro-Picado 2019) and fans’ consumption intentions (Navarro-Picado et al. 2020). This relationship needs further understanding, as perceived organizational legitimacy could act through trustworthiness and the justifiability of a specific decision taken. These illustrations and discussions lead to the following two hypotheses:

#### **H**_**1**_

Higher levels of perceived organizational legitimacy would increase the leagues’ trustworthiness.

#### **H**_**2**_

Higher levels of perceived organizational legitimacy would increase the justifiability of decisions.

### Trustworthiness during a crisis

Despite being an essential variable for decision-making (Pena-Marin and Wu 2019), trustworthiness has rarely been studied in the context of sport management. Trustworthiness is a multidimensional construct composed of ability, benevolence, and integrity (Mayer and Davis 1999). Ability is considered the most tangible dimension (Jacobsen and Andersen 2015), representing the intellect and interpersonal skills needed for successful performance (Maxwell and Lévesque 2014). Benevolence accounts for the perception that the supplier would place the consumer’s benefit before its own interests (Caldwell and Hayes 2007), becoming the most emotional dimension of the construct (Moloney 2005). The last dimension is integrity, which is more related to ethical requirements (Caldwell and Hayes 2007) and the perceived expectancy of organizations keeping their words and fair treatments (Hosmer 1995).

Reliance is an important variable that can strengthen an organization’s relationships with stakeholders (Bejou et al. 1998; Crane 2020). It is built from a cognitive process (Morrow et al. 2004; Ozdemir et al. 2020) that involves the experience of individuals with the organization (Lovell 2009) and the reputation of the organization as to whether it is honorable (Flores and Solomon 1998; Bellucci and Park 2020). Trustworthiness captures the tendency or propensity to believe in something (Sekhon et al. 2014) and is considered a common antecedent of reliance itself (Caldwell and Clapham 2003). Consequently, the following hypothesis is formulated:

#### **H**_**3**_

Higher levels of trustworthiness would increase the reliance on a sport league.

### Level of reliance in sport leagues

Reliance is a relevant construct to consider during difficult time periods (Rousseau et al. 1998). People tend to believe that the organizations they rely on would solve problems in the best way for their customers (Martínez et al. 2020). Specifically, within a sports context, reliance serves as an enhancer of fans’ consumption behaviors (Kim et al. 2011). To better understand the relevance of reliance in the context of the COVID-19 pandemic, it is valuable to follow Rousseau et al.’s (1998) definition. It suggests that reliance is a “psychological state comprising the intention to accept vulnerability based upon positive expectations of the intentions or behavior of another” (p. 395). Apparently, this explanation would imply accepting vulnerability in a moment in which people are already vulnerable due to the pandemic. The main difference between these terms is that trustworthiness is related to the reputation managed by the trustee (Sekhon et al. 2014), while reliance is based on subjective perception, which acts as an individual lens for each person (Caldwell and Clapham 2003).

The GBL and the EPL are visible due to historical successes and attract high consumption levels through multimedia (Ajadi et al. 2020). As with any other sport institution, they are expected to rely on fans (Garbarino and Johnson 1999) and thus benefit from higher levels of fan consumption intentions (Cho et al. 2020) under the assumption that any related decision can be justified and would not substantially hamper the existent level of reliance (Alonso 2016). These discussions lead to the following hypotheses:

#### **H**_**4**_

Higher levels of reliance would positively impact the multimedia consumption intention.

#### **H**_**5**_

Higher levels of reliance would positively impact the justifiability of the decision.

### Justifying unconventional decisions

According to NT, individuals tend to establish an anchor toward what is considered normal to themselves, making it easier to justify sticking to some actions instead of switching to alternatives (Kahneman and Miller 1986). NT originally suggested that the status quo is generally considered “more normal” than an alternative situation. However, recent research findings indicate that this could be reversed in cases where past behavior, situational expectations, and social norms advise a switching decision as more normal than a staying decision (Feldman 2020). When evaluating what is normal and what is not, individuals are more affected by abnormal decisions, arising sentiments of regret (Feldman et al. 2020). Nonetheless, contextual situations have a relevant impact on the evaluation of whether action or inaction should be considered a comparatively more normal decision (Temerak and El-Manstrly 2019). If individuals tend to favor highly justifiable alternatives, it can be argued that individuals would consume the multimedia of sport organizations perceived to have made justifiable decisions. Accordingly, the following hypothesis was developed:

#### **H**_**6**_

Higher levels of the justifiability of the decision would increase the intention to consume multimedia.

Globalization has impacted almost every person on the planet, which is especially highlighted in times of crisis (Palttala and Vos 2011). Individuals and societies struggle during these scenarios, while different public and private organizations compete for limited resources in an environment that demands rapid learning and actions (Ödlund 2010). During crisis conditions, organizations regularly show poor efficiency and cooperation behaviors (Ödlund 2010) and difficulties in communication (Palttala and Vos 2011). This is why individuals tend to better evaluate institutions that, in these circumstances, show determination toward adaptation (Feldman 2020). Reasonably, in a situation such as the public health crisis derived from the COVID-19 pandemic, individuals would tend to justify some decisions that would normally be considered abnormal and consequently allocate higher value to those organizations that attempt to change rather than remain still. With the justifiability of the decision to be a comparison variable (van de Calseyde et al. 2018), a contrast between the status quo and an adaptation decision arises as a logical test.

**Fig. 1:**
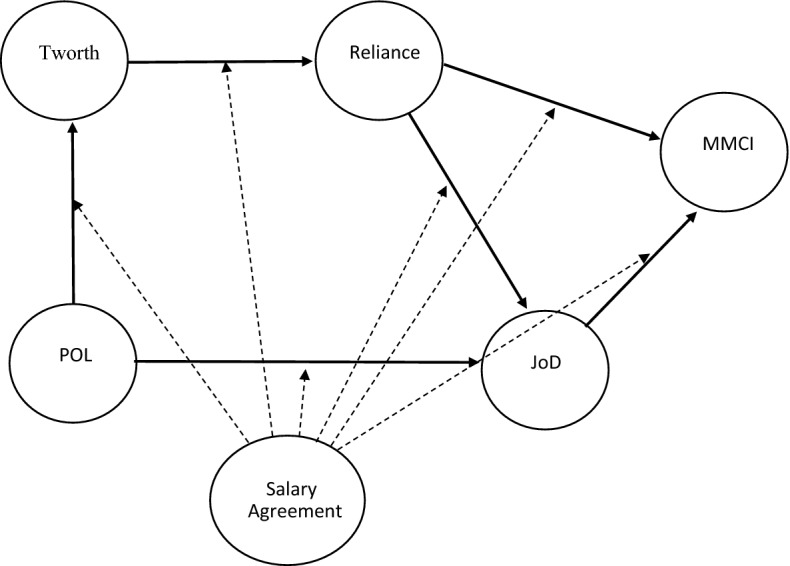
Hypothetical Research Model Depicting the Relationships among Perceived Organizational Legitimacy, Trustworthiness, Reliance, Justifiability of Decision and Multimedia Consumption Intention, moderated by a (no) salary agreement during the COVID-19 crisis. *Note*: dashed line (---) crosses hypothesis affected by moderation. Tworth = Trustworthiness, POL = Perceived Organizational Legitimacy, JoD = Justifiability of Decision, MMCI = Multimedia Consumption Intention

In the context of a pandemic, it can be expected that adaptable sport leagues are likely to make some arrangements to prevail during the crisis period. Even if the organization’s stability has not been jeopardized, social evaluation could positively or negatively impact a sport league depending on the decisions taken (Verbruggen and Van Emmerik 2020). Under these conditions, it can be argued that professional sport leagues who reach a salary agreement during the crisis period would experience an increase in their perceived organizational legitimacy, which would positively impact both the level of perceived trustworthiness and the justifiability of the decision (Fan and Wu 2019). Assuming a domino effect, we expect that, in the same context, lower levels of trustworthiness will be needed to rely on organizations that reach a salary agreement. Similarly, individuals would need lower levels of reliance to justify the decision taken and show higher levels of multimedia consumption intention (Sen and Morwitz 1996) while needing less justifiability to increase their multimedia consumption intention. The following hypotheses were formulated (Fig. 1):

#### **H**_**7a**_

Sport leagues that reached a salary agreement would experience a stronger relationship between perceived organizational legitimacy and trustworthiness.

#### **H**_**7b**_

Those sport leagues that reached a salary agreement would experience a stronger relationship between perceived organizational legitimacy and justifiability of decision.

#### **H**_**7c**_

Those sport leagues that reached a salary agreement would experience a weaker relationship between perceived trustworthiness and reliance.

#### **H**_**7d**_

Those sport leagues that reached a salary agreement would experience a weaker relationship between reliance and justifiability of decision.

#### **H**_**7e**_

Those sport leagues that reached a salary agreement would experience a weaker relationship between reliance and MMCI.

#### **H**_**7f**_

Those sport leagues that reached a salary agreement would experience a weaker relationship between justifiability of decision and MMCI.

## Method

### Participants

Respondents were recruited through either a Qualtrics mailing list or MTurk for Latin American football consumers between April and May 2020. All research participants were over 18 years old and were confirmed to have a fundamental knowledge of football and familiarity with top-tier professional football leagues. After eliminating incomplete questionnaires, 503 valid responses (13 Qualtrics, 490 MTurk) were formally collected. The response ratio was 3.2% in the case of the Qualtrics mailing list (13/407), while MTurk was 58.7% (490/835), with an average age of 30.34 (SD = 10.83). Of the participants, 329 were men, and 174 were women. Table 1 presents the descriptive statistics of the respondents.

**Table 1:** Descriptive statistics of the sample

Gender/age	18–25	26–35	36–45	46-older
Women	73	55	31	15
Men	134	115	49	31

For top-tier professional football leagues, broadcasting and media income represent up to 66% of their income, whereas live event spectator revenue (e.g., ticketing and concessions) is lower. This is the case for both GBL (Horky 2021) and EPL (Kennedy and Kennedy 2021). According to Horky (2021) and Kennedy and Kennedy (2021), income share due to matchday spectators barely represents 13% for football, while other sports within Germany, such as ice hockey (32%), are more dependent on spectator attendance at the game events. Therefore, we chose to conduct the study in a Latin American setting based on the following three considerations: (a) high exposure to and involvement of Latin American sport fans in football; (b) their general high-level knowledge of football and familiarity with top-tier professional football leagues in the world (Giulianotti and Robertson 2012); (c) strong intention of investigators of this study to mitigate any localness effects associated with the EPL and GBL (Mandler et al. 2020).

### Measurement

A questionnaire was developed that contained the following sections: (a) an informed consent, which requested participants to confirm their voluntary participation and that they were above 16 years old and had some knowledge of top-tier football; (b) a little contextualization on COVID-19; its impact on sports and approach differences between the EPL and GBL in these circumstances; (c) the core questionnaire. All measures in this study were adopted or adapted from those developed in previous studies. MMCI was assessed through a three-item scale based on the research of Kim et al. (2011), which includes questions such as *“I will watch or listen to the (Bundesliga/Premier League) games through the media (e.g., TV, Internet, Radio, *etc*.).”* Justifiability of Decision (JoD) is also a three-item scale, based on Inman and Zeelenberg’s (2002) scale, with items such as *“How justifiable is the decision to (not) reach a salary agreement?”* The scale for assessing reliance is based on the research of Kim et al. (2020) and includes items such as *“I would be comfortable giving the (Bundesliga/PremierLeague) total responsibility of the activities.”* Trustworthiness was assessed through a 17-item scale based on the research of Mayer and Davis (1999), including items such as *“The (Bundesliga/Premier League) is capable of performing its job.”* Perceived organizational legitimacy was assessed based on Fidan and Balci’s (2018) scale, which included items such as *“The (Bundesliga/Premier League) is compatible with the values of the general public.”* For sample description purposes, a section on sociodemographic background variables was included in the survey form, asking for the age and sex of the participants.

As the aforementioned scales adapted for this study were originally published in English language scholarly journals, items of each scale were translated into Spanish. Double translations were made to ensure linguistic validity (Chapman and Carter 1979). A panel of five experts with a specialized background in professional sport league management theories and practices were invited to evaluate content validity (Crocker and Algina 1986). The panel members were asked to examine each item’s relevance, representativeness, and clarity for a specific concept. Adopting a standard of 80% agreement among the panel members, all items were retained after revisions and modifications were made to improve linguistic expressions for a few items. All the items were phrased on a Likert 7-point scale, ranging from 1 = strong disagreement to 7 = strong agreement. The items were arranged in a random order within each of the sections, and Table 2 shows the means and standard deviations for all items.

**Table 2:** Descriptive statistics for predictive and criterion variables

Construct	Variable	Item	Mean	SD
Perceived Organizational Legitimacy	ConLeg_01	The (Bundesliga/Premier League) is compatible with the values of the general public	4.73	1.62
	ConLeg_02	The general public believes that the (Bundesliga/Premier League) are well trained	5.09	1.38
	ConLeg_03	The (Bundesliga/Premier League) is well-equipped to meet the general public's expectations	5.02	1.40
	ProLeg_01	The (Bundesliga/Premier League) meets the standards set by legal regulations (laws, curricular, directives, etc.) in its operating procedures (rules, practices, methods, etc.)	5.14	1.40
	ProLeg_02	The (Bundesliga/Premier League) rigorously follow legal regulations	5.01	1.39
	StrLeg_01	The structure of the (Bundesliga/Premier League) is designed to meet the standards required by the sports industry	5.18	1.40
	StrLeg_02	The (Bundesliga/Premier League) has appropriate units (administration, marketing, etc.) to accomplish its goals	5.39	1.26
	StrLeg_03	The (Bundesliga/Premier League) is structured to ensure accomplishing organizational goals	5.29	1.27
Trustworthiness	AbiDe_01	The (Bundesliga/Premier League) is very capable of performing its job	5.40	1.33
	AbiDe_02	The (Bundesliga/Premier League) is known to be successful at the things it tries to do	5.27	1.28
	AbiDe_03	The (Bundesliga/Premier League) has much knowledge about the work that needs to be done	5.16	1.34
	AbiDe_04	I feel very confident about the (Bundesliga/Premier League) skills	5.02	1.35
	AbiDe_05	The (Bundesliga/Premier League) has specialized capabilities that boosts its performance	5.10	1.26
	AbiDe_06	The (Bundesliga/Premier League) is well qualified	5.24	1.30
	BenDe_01	The (Bundesliga/Premier League) is very concerned about its members welfare	4.95	1.57
	BenDe_02	Members’ needs and desires are very important to the (Bundesliga/Premier League)	4.96	1.50
	BenDe_03	The (Bundesliga/Premier League) would not knowingly do anything to hurt its members	4.84	1.55
	BenDe_04	The (Bundesliga/Premier League) really looks out for what is important to its members	5.03	1.41
	BenDe_05	The (Bundesliga/Premier League) will go out of its way to help its members	4.95	1.47
	IntDe_01	The (Bundesliga/Premier League) has a strong sense of justice	4.82	1.51
	IntDe_02	I never have to wonder whether the (Bundesliga/Premier League) will stick to its word	4.39	1.52
	IntDe_03	The (Bundesliga/Premier League) tries hard to be fair in dealings with others	4.91	1.32
	IntDe_04	The (Bundesliga/Premier League) actions and behaviors are consistent	5.01	1.34
	IntDe_05	I like the (Bundesliga/Premier League) values	4.89	1.55
	IntDe_06	Sound principles seem to guide the (Bundesliga/Premier League) behavior	4.85	1.43
Reliance	Relia_01	I would rely on the (Bundesliga/Premier League) without hesitation	4.25	1.73
	Relia_02	I think following the (Bundesliga/Premier League will lead to positive outcomes	4.45	1.56
	Relia_03	I would feel comfortable relying on the (Bundesliga/Premier League) in the future	4.55	1.49
	Relia_04	When the task was hard, I feel like I could depend on the (Bundesliga/Premier League)	4.14	1.66
	Relia_05	If I were facing a very hard task in the future, I would want to have the (Bundesliga/Premier League) with me	4.27	1.66
	Relia_06	I would be comfortable allowing the (Bundesliga/Premier League) to make all decisions	3.89	1.79
	Relia_07	If I could not make a decision, I would allow the (Bundesliga/Premier League) to make it for me	3.54	1.91
	Relia_08	I would be comfortable giving the (Bundesliga/Premier League) total responsibility of the activities	3.73	1.87
	Relia_09	I really believe there is no need to monitor the route decisions of the (Bundesliga/Premier League)	4.12	1.79
	Relia_10	I would be comfortable allowing the (Bundesliga/Premier League) to take a decision, even if I could not monitor it	3.66	1.89
Justifiability of Decision	JoD_01	How justifiable is the decision to (not) reach a salary agreement?	4.61	1.67
	JoD_02	How easy to defend is the decision to (not) reach a salary agreement?	4.33	1.62
	JoD_03	How logical is the decision to (not) reach a salary agreement?	4.69	1.47
Multimedia Consumption Intention	MMCI_01	I will track the news on the (Bundesliga/Premier League) through the media (e.g. TV, Internet, Radio, etc.)	4.69	1.70
	MMCI_02	I will watch or listen to the (Bundesliga/Premier League) games through the media (e.g. TV, Internet, Radio, etc.)	4.79	1.77
	MMCI_03	I will support the (Bundesliga/Premier League) by watching or listening to the games through the media (e.g. TV, Internet, Radio, etc.)	4.58	1.71

### Procedures

We aimed to assess the moderating effect of the perceived differences between what was done by the Bundesliga and the Premier League during the health crisis. A little contextualization was articulated and presented to all participants at the beginning of the survey. This was aligned with the strong speculations by major media outlets regarding the lack of interest in making a change on the part of the EPL (Roan 2020) and was also consistent with the scientific literature with reference to the undeserving efforts made by this league (Kennedy and Kennedy 2021). Textually, this introduction said, “*Given the current global contingency, some sports leagues, unlike others, have taken important measures to face these difficult times. For example, the German Bundesliga has actively collaborated so that all its clubs reach salary agreements and maintain the jobs of the players and the entire staff. Nevertheless, little effort has been made to achieve these goals in the EPLeague.*”

Responses to the questionnaire were obtained electronically through Qualtrics mailing distribution; individuals received an email invitation to participate and a link to the survey or MTurk for Latin America through Amazon´s MTurk platform. After individuals agreed to participate in the study, they were randomly assigned to respond to the questionnaire regarding only one league, Bundesliga (261 respondents) or Premier League (242 respondents). As the study was conducted in a different sociocultural, linguistic, and sports setting than the original scales in the questionnaire, it was deemed necessary to reexamine the validity and reliability of the measures in the questionnaire. An exploratory factor analysis (EFA) was performed using the SPSS program, followed by a confirmatory factor analysis (CFA) by executing the AMOS program (Anderson and Gerbing 1988). Structural equation model (SEM) analyses were conducted to examine the research hypotheses.

## Results

When conducting EFA, a factor loading threshold was set to 0.50 or higher without double-loading, while a criterion of 0.70 was set for a Cronbach’s alpha coefficient (Nunnally 1994). All measures satisfied these criteria; subsequently, it was deemed appropriate to proceed with conducting a CFA. The model fit indexes included RMSEA = 0.064, CFI = 0.908, IFI = 0.909, TLI = 0.899, and normed chi-square of 3.05, indicating that the data fit the measurement model well. All items were preserved because of their high levels of convergence and R^2^. To avoid any possible underestimation from the Cronbach’s alpha (Smith 1974), a composite reliability test was conducted (Jöreskog 1971), setting the threshold to the recommended 0.65 (Steenkamp and Geyskens 2006). Adopting the threshold of 0.50, convergent validity was confirmed by calculating average variance extracted (AVE) coefficients (Fornell and Larcker 1981). Finally, discriminant validity was tested by verifying that number 1 was not included in the confidence interval of any correlation pair (Anderson and Gerbing 1988). All these findings suggest adequate psychometric properties of the measurement scales, and their respective figures are shown in Table 3 regarding the CFA, composite reliability, and convergent validity. Table 4 refers to the correlational analysis and discriminant validity.

**Table 3:** Confirmatory factor analysis, composite reliability, and convergent validity

Item	Standardize solution	t-value	Item R^2^	Factor	Cronbach’s Alpha	Composite Reliability	AVE
ConLeg_01	0.756	65.522	0.547	Consequential legitimacy	0.832	0.841	0.638
ConLeg_02	0.830	82.675	0.711				
ConLeg_03	0.809	80.571	0.661				
ProLeg_01	0.841	82.063	0.715	Procedural legitimacy	0.844	0.844	0.729
ProLeg_02	0.867	80.894	0.744				
StrLeg_01	0.830	82.887	0.689	Structural legitimacy	0.849	0.849	0.653
StrLeg_02	0.770	95.994	0.593				
StrLeg_03	0.823	93.186	0.679				
AbiDe_01	0.800	91.350	0.641	Ability	0.891	0.890	0.574
AbiDe_02	0.728	92.528	0.527				
AbiDe_03	0.711	86.545	0.504				
AbiDe_04	0.797	83.163	0.635				
AbiDe_05	0.726	90.519	0.529				
AbiDe_06	0.780	90.317	0.608				
BenDe_01	0.872	70.534	0.758	Benevolence	0.919	0.922	0.703
BenDe_02	0.878	74.108	0.772				
BenDe_03	0.708	70.176	0.502				
BenDe_04	0.864	79.972	0.745				
BenDe_05	0.857	75.693	0.736				
IntDe_01	0.848	71.629	0.721	Integrity	0.890	0.894	0.588
IntDe_02	0.577	64.967	0.334				
IntDe_03	0.698	83.442	0.491				
IntDe_04	0.785	83.978	0.616				
IntDe_05	0.820	70.686	0.669				
IntDe_06	0.836	76.273	0.697				
Relia_01	0.782	55.144	0.611	Reliance	0.935	0.936	0.599
Relia_02	0.570	64.104	0.324				
Relia_03	0.706	68.404	0.498				
Relia_04	0.812	55.823	0.657				
Relia_05	0.761	57.128	0.579				
Relia_06	0.844	48.800	0.713				
Relia_07	0.872	41.510	0.762				
Relia_08	0.888	44.700	0.790				
Relia_09	0.651	51.536	0.425				
Relia_10	0.797	43.418	0.635				
JoD_01	0.911	62.089	0.831	Justifiability of decision	0.879	0.879	0.709
JoD_02	0.767	59.880	0.588				
JoD_03	0.842	71.459	0.710				
MMCI_01	0.816	62.017	0.665	Multimedia consumption intention	0.897	0.898	0.746
MMCI_02	0.895	60.705	0.800				
MMCI_03	0.878	60.176	0.772				

All t-values significant at .01 level

*ConLeg* consequential legitimacy, *ProLeg* procedural legitimacy, *StruLeg* structural legitimacy, *AbiDe* ability (trustworthiness), *BenDe* benevolence (trustworthiness), *IntDe* integrity (trustworthiness), *Relia* reliance, *JoD* justifiability of decision, *MMCI* multimedia consumption intention

**Table 4:** Correlational analysis and discriminant validity

Pair of constructs	Correlation	SEE	Confidence interval	
Consequential Leg. ↔ Procedural Leg.	0.772	0.038	0.701	0.828
Consequential Leg. ↔ Structural Leg.	0.797	0.040	0.716	0.856
Consequential Leg. ↔ Ability	0.854	0.027	0.800	0.887
Consequential Leg. ↔ Benevolence	0.724	0.037	0.652	0.783
Consequential Leg. ↔ Integrity	0.762	0.033	0.706	0.817
Consequential Leg. ↔ Reliance	0.462	0.046	0.378	0.528
Consequential Leg. ↔ Justifiability of Dec.	0.712	0.038	0.636	0.770
Consequential Leg. ↔ MMCI	0.457	0.050	0.370	0.538
Procedural Leg. ↔ Structural Leg.	0.782	0.043	0.699	0.842
Procedural Leg. ↔ Ability	0.757	0.041	0.689	0.821
Procedural Leg. ↔ Benevolence	0.712	0.036	0.659	0.777
Procedural Leg. ↔ Integrity	0.740	0.033	0.682	0.794
Procedural Leg. ↔ Reliance	0.390	0.044	0.309	0.457
Procedural Leg. ↔ Justifiability of Dec.	0.661	0.038	0.599	0.724
Procedural Leg. ↔ MMCI	0.334	0.047	0.255	0.416
Structural Leg. ↔ Ability	0.931	0.021	0.894	0.963
Structural Leg. ↔ Benevolence	0.694	0.042	0.627	0.756
Structural Leg. ↔ Integrity	0.698	0.036	0.635	0.749
Structural Leg. ↔ Reliance	0.310	0.046	0.229	0.383
Structural Leg. ↔ Justifiability of Dec.	0.529	0.045	0.447	0.599
Structural Leg. ↔ MMCI	0.406	0.046	0.334	0.486
Ability ↔ Benevolence	0.748	0.037	0.677	0.806
Ability ↔ Integrity	0.787	0.034	0.728	0.835
Ability ↔ Reliance	0.451	0.042	0.374	0.514
Ability ↔ Justifiability of Dec.	0.587	0.043	0.505	0.651
Ability ↔ MMCI	0.543	0.039	0.478	0.609
Benevolence ↔ Integrity	0.930	0.014	0.902	0.949
Benevolence ↔ Reliance	0.556	0.040	0.490	0.621
Benevolence ↔ Justifiability of Dec.	0.744	0.031	0.691	0.793
Benevolence ↔ MMCI	0.390	0.052	0.301	0.472
Integrity ↔ Reliance	0.671	0.038	0.603	0.728
Integrity ↔ Justifiability of Dec.	0.780	0.029	0.728	0.821
Integrity ↔ MMCI	0.495	0.047	0.418	0.570
Reliance ↔ Justifiability of Dec.	0.520	0.045	0.438	0.587
Reliance ↔ MMCI	0.630	0.035	0.571	0.681
Justifiability of Dec. ↔ MMCI	0.328	0.052	0.240	0.415

*Leg.* legitimacy, *Dec.* decision, *MMCI* multimedia consumption intention

**Fig. 2:**
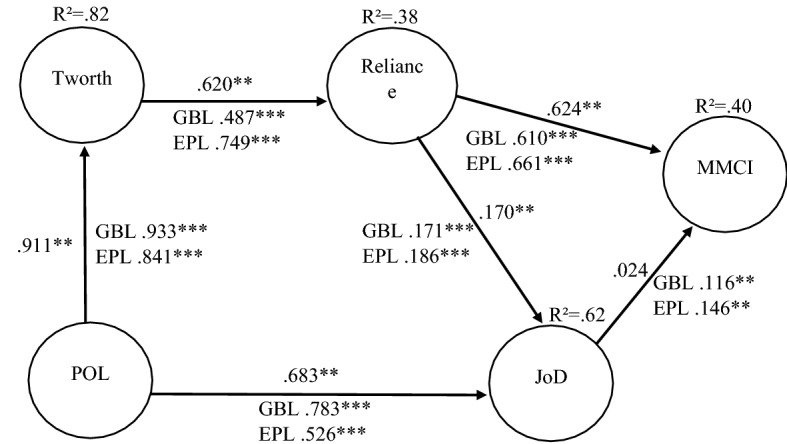
Structural Equation Model Examining the Relationships among Perceived Organizational Legitimacy, Trustworthiness, Reliance, Justifiability of Decision and Multimedia Consumption Intention. *Note*: *p < .10 **p < .05 ***p < .01. Tworth = Trustworthiness, POL = Perceived Organizational Legitimacy, JoD = Justifiability of Decision, MMCI = Multimedia Consumption Intention. Moderation probability: POL → Tworth .268, POL → JoD .000***, Tworth → Reliance .001***, Reliance → Jod .000***, Reliance → MMCI .202, JoD → MMCI .095*

The SEM analyses revealed that satisfactory values support model fit to the data, as the model achieves an RMSEA of 0.071, CFI of 0.883, IFI of 0.883, TLI of 0.874, and normed chi-square of 3.54. The findings indicate that perceived organizational legitimacy had a positive effect on trustworthiness (.911, *p* < .01) and justifiability of decision (.683, *p* < .01), supporting H_1_ and H_2_. Trustworthiness positively impacted reliance (.620, *p* < .01), while reliance had a positive effect on justifiability of decision (.170, *p* < .01) and MMCI (.624, *p* < .01), supporting H_3_, H_4_, and H_5_. At this step of the study, the relationship between the justifiability of decision and MMCI was not found to be statistically significant (*p* > .51) (Fig. 2).

During the data collection process, participants were randomly assigned to evaluate the decision of either the Bundesliga (reaching an agreement) or the Premier League (no agreement). Therefore, and in line with NT, the Bundesliga took action while the Premier League stayed in its status quo. A multi-sample analysis was performed to test the moderation effect, which corresponded to H_7a_ through H_7f_. A total of 261 individuals were assigned to Bundesliga, while 242 were allocated to the Premier League. A CFA invariance test was performed prior to the multi-sample analysis (Silva et al. 2019). It has been recommended that three test levels should be assessed: (a) configural invariance (good model fit in the multi-group sample); (b) metric invariance (unconstrained vs. structural weights CFI difference test below .01); (c) structural invariance (unconstrained vs. structural weight chi-square insignificance differences) (Cheung and Rensvold 2009). Configural invariance was supported with coefficients of RMSEA = 0.049, CFI = 0.887, IFI = 0.888, TLI = 0.875, and normed chi-square = 2.20. Metric invariance was satisfactory, with a CFI difference of 0.000 (unconstrained 0.887–0.887 measurement). The chi-square difference test was unsatisfactory. Nonetheless, we assumed the invariance as two out of three tests were accomplished and sticking to the suggestion made by Cheung and Rensvold (2009) regarding the lack of practicality and restrictiveness of the chi-square difference test, which was also recently backed by other researchers (French and Finch 2006). A multi-sample analysis allowed us to recognize the disparities between the coefficients of the two models by generating a single solution for each of them (Table 5).

**Table 5:** Multi-sample analysis—action (Bundesliga) versus inaction (Premier League)

Constraint	Estimated coefficient (Bundesliga)	Estimated coefficient (Premier League)	(d.f) *X*^2^ differences	Probability
POL → Tworth	.933***	.841***	(1) 1.227	.268
POL → JoD	.783***	.526***	(1) 23.584	.000***
Tworth → Reliance	.487***	.749***	(1) 10.540	.001***
Reliance → JoD	.171***	.186***	(1) 36.546	.000***
Reliance → MMCI	.610***	.661***	(1) 1.624	.202
JoD → MMCI	.116**	.146**	(1) 2.795	.095*

*POL* perceived organizational legitimacy, *Tworth* trustworthiness, *JoD* justifiability of decision, *MMCI* multimedia consumption intention

*p < .10 **p < .05 ***p < .01

As shown in Table 5, there were no significant differences between perceived organizational legitimacy and trustworthiness. However, there was a strong significance in the differences between perceived organizational legitimacy and justifiability of decision, with a higher coefficient in the agreement (Bundesliga) case. There was also strong significance when analyzing the differences between trustworthiness and reliance, and from reliance to the justifiability of decision, showing higher coefficients for the no-agreement (Premier League) group. The reliance on the MMCI string appears to be insignificant, whereas the string of justifiability of decision to MMCI showed a trend of significance, with a higher coefficient for the no-agreement (Premier League) group. These findings revealed that in the context of the COVID-19 crisis, higher levels of perceived organizational legitimacy were allocated to leagues that reached an agreement and therefore increased the justifiability of this decision.

Additionally, for cases in which an agreement was reached, lower levels of trustworthiness were needed to rely on that league. Simultaneously, lower levels of reliance were required to manifest a multimedia consumption intention. Finally, less justifiability was needed from decisions taken by sport leagues who reached an agreement to have fans consuming multimedia.

## Discussion

Drawing on the social judgment theory and norm theory, this study developed a model to investigate whether fans’ judgment toward decisions made by sport leagues is aligned with theoretical expectations. Our main goal was to distinguish the evaluation differences made by fans concerning the efforts made by the sport leagues to reach an agreement versus maintaining the status quo. This was done during the global health crisis of COVID-19 and examined how this could impact the multimedia consumption intention of those leagues.

Multimedia consumption is deemed a relevant variable for top-tier leagues as broadcasting represents up to 66% of the leagues’ budget (Horky 2021), especially during the lockdown period when fans are not allowed to access the arenas physically (Corsini et al. 2020; Majumdar and Naha 2020). However, fans who were directly impacted by the shutdown may experience a localness bias during that period (Mandler et al. 2020). Overall, the Latin American region has a considerably knowledgeable (Ridge 2017) and loyal fan base (Sotomayor 2020) that consumes excessive multimedia football content, particularly because of local lockdown policies caused by the pandemic crisis.

Aligned with the social judgment theory, our findings suggest that higher levels of perceived organizational legitimacy derive higher levels of trustworthiness and justifications of decisions. Therefore, legitimacy once again appears as a relevant input for organizational success, as our findings suggest that it is easier for individuals to depend (rely) on and justify decisions taken by organizations that enjoy higher levels of perceived legitimacy.

However, and aligned with recent findings of the norm theory, individuals seem to appreciate that GBL reached a downgrade salary agreement—what should normally be considered normal (status quo) is poorly evaluated during a crisis period. This paper advances the knowledge on norm theory, as it argues that in a sports context and during a crisis period, evaluations made by individuals are reversed to those expected during “normal” periods. Specifically, the fact that EPL did not intervene in salary agreements (downward) was perceived as acting the wrong way by doing nothing. Conversely, the GBL that actively worked on salary reductions (not normal) was perceived as positive actors, ultimately enjoying higher levels of consumption intention.

Decisions taken by trusted institutions appear as easier to justify; simultaneously, both reliance and justifiability derive into higher intentions to consume multimedia products from the sport leagues. This may be the case if fans evaluate a lack of action by the league as jeopardizing the continuity (Rousseau et al. 1998) of the sports activity, representing higher risk of losing this entertainment source in a vulnerable time. These findings suggest that fans’ judgments and evaluations would stick to a legitimacy baseline but simultaneously demand rapid adaptation to circumstances. Specifically, the fact that perceived organizational legitimacy toward trustworthiness shows no significant difference between the evaluated leagues argues that legitimacy is a baseline evaluation tool for fans regardless of the circumstances. However, after the confirmation of the invariance test, our findings suggest that Perceived Organizational Legitimacy → Justifiability of Decision, Trustworthiness → Reliance, Reliance → Justifiability of Decision, and Justifiability of Decision → Multimedia Consumption Intention strings are indeed significant. These findings indicate that specific contextual decisions are impacted by a more dynamic evaluation process made by the fans, which in turn would mean that, in a “new normality” context, international broadcasting audiences could tend to favor sport leagues who make quick adjustments and show an adaptation interest.

From a strategic management perspective, these findings argue that fans are sensible to actions taken during crisis periods, and attention toward fans’ evaluation demands may be crucial. This is a relevant finding. For example, clubs are struggling with the lack of gate receipts, which represents the best way to attract sponsors, sign broadcasting contracts, and deliver their exposure to fans. It also argues that regardless of the situation or context, legitimacy is a foundation block for fans’ judgment and a powerful tool for sport institutions that could be used/encouraged through communication strategies.

This study has a limitation in that it only considers two top leagues of a specific sport. Although football is a globally recognized sport, the limitation of this single-sport analysis should be considered when interpreting the findings. Although practical and efficient for data collecting, the fact that most of our research responses were obtained through Amazon’s MTurk should arise as a tentative limitation (Aguinis et al. 2021). Nevertheless, due to the health crisis of the pandemic, it represented an optimal data collection option for the current investigation. Finally, readers should consider that the sample consists of international fans, therefore not all results are transferable to domestic fans. Proximity to the source and localness bias could affect the impact on fans and therefore additional research should be done to this type of consumers. Future research should consider the analysis of different sports or less relevant leagues, where localness or a preconceived smaller resource availability could arouse different responses. Therefore, replication of this research is recommended for different sport scenarios and nationalities.
